# Comprehensive Study of Antibiotic Resistance in *Enterococcus* spp.: Comparison of Influents and Effluents of Wastewater Treatment Plants

**DOI:** 10.3390/antibiotics13111072

**Published:** 2024-11-11

**Authors:** Ji-Hyun Park, Kyung-Seon Bae, Jihyun Kang, Eung-Roh Park, Jeong-Ki Yoon

**Affiliations:** 1 Han River Environment Research Center, National Institute of Environment Research, Yangpyeong-gun, Incheon 12585, Gyeonggi-do, Republic of Korea; 2 Division of Water Supply and Sewerage Research, National Institute of Environment Research, Incheon 22689, Gyeonggi-do, Republic of Korea; baeks1207@korea.kr (K.-S.B.); jhkang2@korea.kr (J.K.); erpark@korea.kr (E.-R.P.); jkyun@korea.kr (J.-K.Y.)

**Keywords:** *Enterococcus* spp., antibiotic resistance, biofilm, antibiotic resistance gene, wastewater treatment plant

## Abstract

**Background/Objectives**: The spread of antibiotic resistance, particularly through *Enterococcus* spp., in wastewater treatment plants (WWTPs) poses significant public health risks. Given that research on antibiotic-resistant enterococci and their antibiotic-resistance genes in aquatic environments is limited, we evaluated the role of *Enterococcus* spp. in WWTPs by comparing the antibiotic resistance rates, gene prevalence, biofilm formation, and residual antibiotics in the influent and effluent using culture-based methods. **Methods**: In 2022, influent and effluent samples were collected from 11 WWTPs in South Korea. Overall, 804 *Enterococcus* strains were isolated, and their resistance to 16 antibiotics was assessed using the microdilution method. **Results**: High resistance to tetracycline, ciprofloxacin, kanamycin, and erythromycin was observed. However, no significant differences in the overall resistance rates and biofilm formation were observed between the influent and effluent. Rates of resistance to ampicillin, ciprofloxacin, and gentamicin, as well as the prevalence of the *tetM* and *qnrS* genes, increased in the effluent, whereas resistance rates to chloramphenicol, florfenicol, erythromycin, and tylosin tartrate, along with the prevalence of the *optrA* gene, decreased. *E. faecium*, *E. hirae*, and *E. faecalis* were the dominant species, with *E. faecalis* exhibiting the highest resistance. **Conclusions**: Our results suggest that WWTPs do not effectively reduce the rates of resistant *Enterococcus* spp., indicating the need for continuous monitoring and improvement of the treatment process to mitigate the environmental release of antibiotic-resistant bacteria.

## 1. Introduction

Antibiotic resistance is an escalating global health crisis driven by the emergence of multidrug-resistant (MDR) bacteria, which pose a significant threat to public health [1]. The excessive use of antibiotics to treat infections applies selective pressure on bacteria, fostering the emergence and persistence of resistant strains. This raises concerns regarding their transmission and survival in various environments [2,3]. The increasing prevalence of antibiotic resistance not only undermines the effectiveness of existing treatments but also complicates efforts to manage and control infections, particularly in healthcare settings, leading to an increase in healthcare-associated infections caused by these hard-to-treat pathogens [4]. Moreover, the impact of antibiotic resistance extends beyond human health and affects agriculture, animal husbandry, and environmental ecosystems [5]. Contamination from pharmaceutical manufacturing, agricultural runoff, and inadequate wastewater treatment fosters the persistence and spread of resistant bacteria in natural water bodies and soils, further compounding this problem [6,7].

*Enterococcus* spp., recognized as opportunistic pathogens, have garnered interest for their ability to acquire and disseminate antibiotic-resistance genes (ARGs). These bacteria are responsible for severe human infections including urinary tract infections, endocarditis, skin infections, and bacteremia [8,9]. *E. faecalis* and *E. faecium*, which constitute over 80% of *Enterococcus* spp. isolates, are major contributors to nosocomial infections, ranking third and fourth globally, after *Staphylococcus aureus* and *Pseudomonas aeruginosa* [10]. *E. faecalis* accounts for 85–90% of these infections, whereas *E. faecium* is responsible for 5–10% of infections [11,12]. Both species are demonstrating a global increase in the prevalence of antibiotic-resistant strains, as reported by Polish and European health agencies [13]. Beyond their clinical significance, enterococci serve as indicators for monitoring fecal contamination in water due to their abundance in the human intestine and their resilience to environmental conditions [14]. However, research on antibiotic-resistant enterococci and their ARGs in aquatic environments remains limited, which is crucial for understanding their spread [15].

Aquatic environments, particularly wastewater treatment plants (WWTPs), serve as critical reservoirs and transmission pathways for antibiotic-resistant bacteria (ARB), facilitating their movement between human and animal populations [16]. WWTPs have been identified as hotspots for the spread of ARB and ARGs, integrating antibiotics from various sources such as households, hospitals, and agriculture [15,17]. These facilities are substantial sources of antibiotic residues, ARB, and ARGs, which are continuously released into the environment, leading to the contamination of surface water, groundwater, and agricultural soil [18,19]. The presence of pharmaceuticals in wastewater exacerbates antibiotic resistance [20]. The urban water cycle, which involves water use and sewage discharge, transfers resistance between the environment and humans [21]. The environmental conditions in WWTPs facilitate bacterial survival and horizontal gene transfer of antibiotic resistance, thereby promoting the spread of ARB across aquatic and terrestrial ecosystems [22].

*Enterococcus* spp. are key members of the microbial communities in WWTPs and are associated with pollution from human and animal waste. MDR *Enterococcus* strains are commonly found in these facilities [23]. They exhibit greater environmental persistence than *Escherichia coli* and are resilient to various environmental stressors [24,25]. Their antibiotic resistance allows them to survive and spread in hospital environments, with transmission occurring from wastewater from domestic, industrial, veterinary, and hospital sources to municipal WWTPs [26]. Enterococci serve as reservoirs for ARGs aided by their conjugative plasmids and transposons, which can transfer resistance to other bacteria [27]. The release of ARB into water bodies can contribute to the spread of antibiotic resistance among native bacterial populations [15].

The widespread presence of *Enterococcus* spp. in water sources poses a health risk to individuals who come into contact with these environments. Therefore, continuous monitoring of enterococci in aquatic environments and identification of hotspots of MDR *Enterococcus* spp. are essential strategies to protect public health. This study aimed to evaluate how *Enterococcus* spp. in WWTPs, which are known hotspots for ARB, contribute to the spread of antibiotic resistance. We compared antibiotic resistance rates, ARG prevalence, biofilm formation, and residual antibiotic levels between the influent and effluent at WWTPs, in addition to resistance rates and gene prevalence among different *Enterococcus* spp. isolated from WWTPs.

## 2. Results

### 2.1. Identification of Enterococcus *spp.*

**Figure 1:**
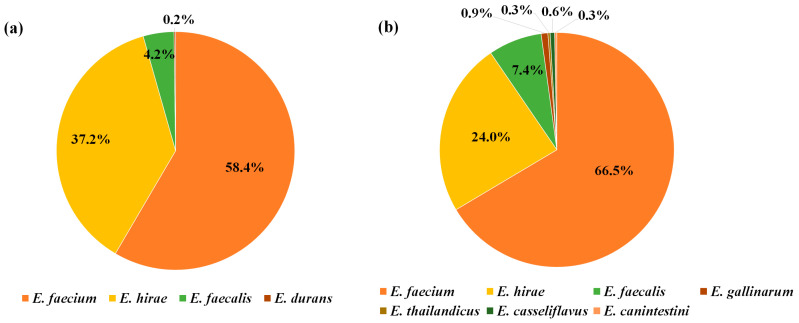
Identification of *Enterococcus* spp. isolated from the wastewater treatment plants (WWTPs). (**a**) Species composition in the influent. (**b**) Species composition in the effluent.

Overall, 804 *Enterococcus* isolates were identified from 11 WWTPs, with 479 and 325 isolates characterized from the influents and effluents, respectively. The distribution of *Enterococcus* spp. in the influent and effluent is presented in Figure 1. Four *Enterococcus* spp. were identified in the influent, with *E. faecium* being the most dominant at 280 isolates (58.5%). This was followed by *E. hirae* with 178 isolates (37.2%), *E. faecalis* with 20 isolates (4.2%), and *E. durans* with one isolate (0.2%). Further, seven *Enterococcus* spp. were identified in the effluent, with *E. faecium* being the most dominant, comprising 216 isolates (66.5%). This was followed by *E. hirae* with 78 isolates (24%), *E. faecalis* with 24 isolates (7.4%), *E. gallinarum* with three isolates (0.9%), and *E. casseliflavus* with two isolates (0.6%). Additionally, one isolate each of *E. thailandicus* and *E. canintestini* were identified. The proportions of *E. faecium* and *E. faecalis* increased in the effluent from 58.5% to 66.5% and 4.2% to 7.4%, respectively, compared with those in the influent, whereas the proportion of *E. hirae* decreased from 37.2% to 24.0%.

### 2.2. Antibiotic Resistance Phenotype 

Antibiotic resistance of the *Enterococcus* isolates was assessed using 16 different antibiotics. The overall resistance rate of the influent isolates was 59.9%. Additionally, 20.9% of the isolates were MDR (resistant to three or more classes of antibiotics). The overall resistance rate of the effluent isolates was slightly higher (62.8%). The proportion of MDR strains in the effluents was 18.2%. The overall antibiotic resistance and MDR rates were not significantly different between the influents and effluents (Table 1). 

**Figure 2:**
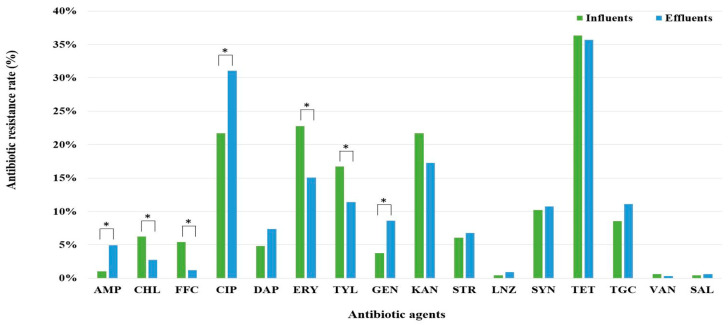
Comparison of resistance rates to 16 antibiotics between *Enterococcus* isolates from influents and effluents of WWTPs. AMP, Ampicillin; CHL, Chloramphenicol; FFC, Florfenicol; CIP, Ciprofloxacin; DAP, Daptomycin; ERY, Erythromycin; TYL, Tylosin tartrate; GEN, Gentamicin; KAN, Kanamycin; STR, Streptomycin; LNZ, Linezolid; SYN, Quinupristin/dalfopristin; TET, Tetracycline; TGC, Tigecycline; VAN, Vancomycin; SAL, Salinomycin. Significant differences between groups are marked with an asterisk (*).

A comparison of the antibiotic resistance rates between the influents and effluents is shown in Figure 2. In the influents, the highest resistance rates were observed in the following order: tetracycline (36.3%), erythromycin (22.8%), ciprofloxacin (21.7%), and kanamycin (21.7%). Conversely, the highest resistance rates observed in the effluents were against tetracycline (35.7%), ciprofloxacin (31.1%), kanamycin (17.2%), and erythromycin (15.1%). Ampicillin, ciprofloxacin, and gentamicin showed a statistically significant increase in resistance rates in the effluents compared with that in the influents, whereas chloramphenicol, florfenicol, erythromycin, and tylosin tartrate showed a decrease. Specifically, ampicillin resistance increased from 1.0% to 4.9%, ciprofloxacin from 21.7% to 31.1%, and gentamycin from 3.8% to 8.6%. In contrast, chloramphenicol resistance decreased from 6.3% to 2.8%, florfenicol from 5.4% to 1.2%, erythromycin from 22.8% to 15.1%, and tylosin tartrate from 16.7% to 11.4%. The resistance rates to other antibiotics were similar between the influents and effluents. 

**Figure 3:**
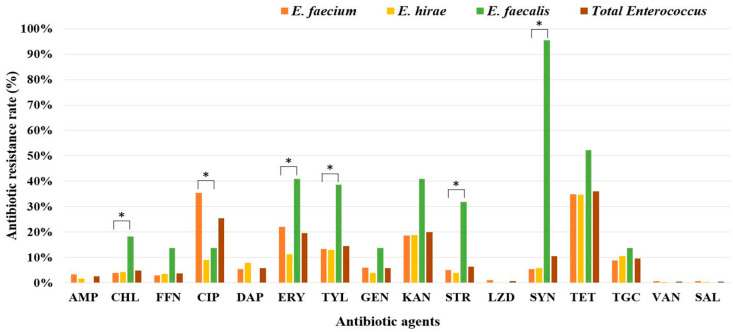
Comparison of resistance rates to 16 antibiotics among the three major *Enterococcus* spp. (*E. faecium*, *E. hirae*, and *E. faecalis*) isolated from the WTTPs. Significant differences between groups are marked with an asterisk (*).

We examined the antibiotic resistance patterns of three major *Enterococcus* spp., *E. faecium, E. hirae*, and *E. faecalis,* isolated from the influents and effluents of the WWTPs (Table 1, Figure 3). Although *E. faecalis* had the lowest number of strains among the three tested species, it exhibited the highest overall antibiotic resistance rate (Table 1). Notably, *E. faecalis* exhibited 100% resistance. Furthermore, 43.2% of the strains were MDR. In contrast, *E. faecium* exhibited resistance and MDR rates of 62% and 20%, respectively, whereas *E. hirae* exhibited resistance and MDR rates of 52% and 15%, respectively. 

The analysis of resistance to 16 different antibiotics revealed that *E. faecalis* generally exhibited higher resistance rates than the overall resistance rates of *Enterococcus* spp. (Figure 3). Notably, the rate of resistance to quinupristin/dalfopristin was the highest, at 95.5%. Other antibiotics against which *E. faecalis* showed significant resistance included tetracycline, kanamycin, erythromycin, tylosin tartrate, streptomycin, chloramphenicol, and florfenicol. In contrast, *E. faecium*, which constituted a majority of the *Enterococcus* isolates, demonstrated a high resistance rate to ciprofloxacin. Further chi-square analysis revealed statistically significant differences in resistance to six antibiotics (chloramphenicol, ciprofloxacin, erythromycin, tylosin tartrate, streptomycin, and quinupristin/dalfopristin) among the different *Enterococcus* spp.

### 2.3. Antibiotic Resistance Genes

**Figure 4:**
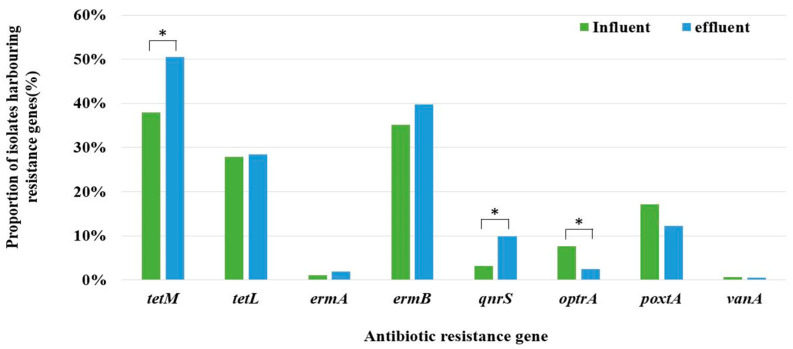
Comparison of the prevalence of antibiotic-resistance genes (ARGs) identified in *Enterococcus* spp. isolated from the influents and effluents of the WTTPs. Significant differences between groups are marked with an asterisk (*).

The presence of ARGs was investigated in 491 of the 804 antibiotic-resistant isolates. Among the 17 ARGs tested, only eight (*tetM*, *tetL*, *ermA*, *ermB*, *qnrS*, *optrA*, *poxtA*, and *vanA*) were identified (Figure 4). In comparison to influent water, the genes *tetM*, *ermB*, and *qnrS* showed an increased prevalence in *Enterococcus* strains isolated from effluent water, whereas the prevalence of the remaining five genes either decreased or remained at comparable levels. The retention rate of *tetM* increased significantly from 38.0% to 50.5%, that of *qnrS* from 3.1% to 9.8%, and that of *optrA* decreased from 7.7% to 2.5%.

Chi-square analysis of the relationship between antibiotic resistance phenotypes and genotypes revealed a statistically significant association between tetracycline resistance and *tetM* and *tetL*. Additionally, a statistically significant relationship was observed between tylosin tartrate resistance and *ermA* and *ermB*.

**Figure 5:**
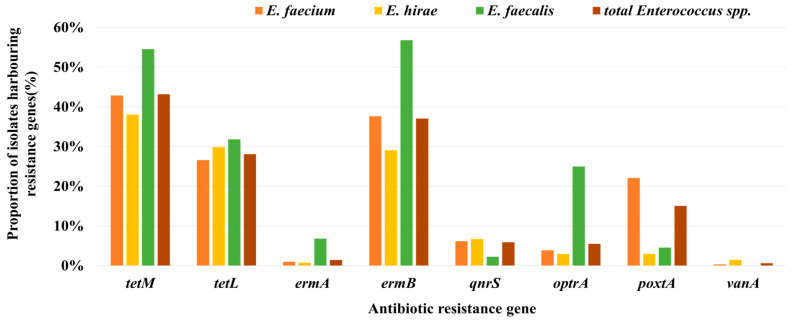
Comparison of the prevalence of ARGs among the three major *Enterococcus* spp. (*E. faecium, E. hirae,* and *E. faecalis*) isolated from the WTTPs.

Analysis of the prevalence of ARGs in the three main *Enterococcus* spp. (*E. faecium*, *E. hirae*, and *E. faecalis*) revealed that *E. faecalis* had the highest overall rate of ARGs (Figure 5). In particular, *tetM*, *tetL*, *ermA*, *ermB,* and *optrA* were found at higher rates in *E. faecalis* than the overall average. Conversely, *poxtA* was found to have a higher prevalence in *E. faecium*.

### 2.4. Evaluation of Biofilm Formation Capability

The comparison of biofilm formation rates between the influents and effluents revealed no significant increase in biofilm formation after passing through the WWTP, with overall formation rates of 23.6% and 20.6%, respectively (Table 2). The evaluation of biofilm formation by the influent isolates indicated weak formation by 18.2%, moderate formation by 5%, and strong formation by 0.4% of isolates. In contrast, weak, moderate, and strong formations were observed in 10.8%, 8%, and 1.8% of the effluent isolates, respectively. A minor increase in the number of strains showing strong formation was noted; however, this difference was not significant.

### 2.5. Measurement of Residual Antibiotics

**Figure 6:**
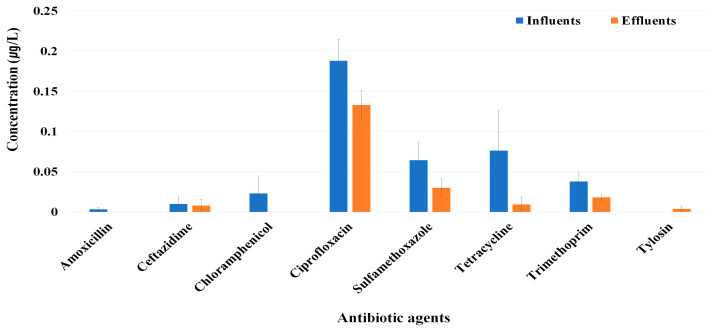
Comparison of residual antibiotic concentrations detected in influents and effluents from the WWTPs.

Measurement of residual antibiotic concentrations for 26 types of antibiotics in influents revealed detectable residues of amoxicillin (0.003 µg/L), ceftazidime (0.01 µg/L), chloramphenicol (0.023 µg/L), ciprofloxacin (0.188 µg/L), sulfamethoxazole (0.064 µg/L), tetracycline (0.076 µg/L), and trimethoprim (0.038 µg/L) (Figure 6). In effluents, residues of ceftazidime (0.008 µg/L), ciprofloxacin (0.133 µg/L), sulfamethoxazole (0.03 µg/L), tetracycline (0.009 µg/L), trimethoprim (0.018 µg/L), and tylosin (0.004 µg/L) were detected. In particular, ciprofloxacin concentrations were notably high in both the influent and effluent water. Overall, the concentrations were observed to decrease in the effluents compared with the influents.

## 3. Discussion

In this study, we conducted a comparative analysis of the antibiotic resistance rates, ARGs, and biofilm formation abilities of *Enterococcus* strains isolated from the influent and effluent of WWTPs. The analysis revealed that despite the acknowledgment of WWTPs as hotspots for ARB and ARG dissemination, the differences between the influent (59.9%) and effluent (62.8%) were not statistically significant. These results indicate a consistent level of antibiotic resistance and the presence of MDR *Enterococcus* strains throughout the wastewater treatment process, suggesting that ARB are not adequately controlled during treatment.

In recent years, a few studies have compared the resistance rates between influents and effluents using culture-based methods. Costa et al. [22] also reported no differences in antibiotic resistance rates between inflows and outflows. The MDR rate of *Enterococcus* spp. in both the influent and effluent of the WWTP was approximately 20%, which was lower than the 68% MDR rate reported for *Enterococcus* spp. in WWTPs in a recent study [26]. This may be due to the lower inclusion of hospital-derived *Enterococcus* strains compared with those in other studies. Additionally, this study revealed that although the overall antibiotic resistance rates remained relatively unchanged before and after wastewater treatment, variations in resistance rates to specific antibiotics were noted. Resistance rates to ampicillin, ciprofloxacin, gentamycin, and tigecycline increased slightly after treatment, whereas those to chloramphenicol, florfenicol, erythromycin, and tylosin tartrate decreased. These findings suggest that while the wastewater treatment process effectively reduces resistance to certain antibiotics, it may inadvertently contribute to increased resistance to others. 

Based on the isolation of *Enterococcus* spp. from the influents and effluents, *E. faecium* was identified as the dominant species, followed by *E. hirae* and *E. faecalis*. Most studies that have focused on WWTPs revealed *E. faecium* to be the dominant species, whereas the proportions of *E. hirae* and *E. faecalis* varied. Typically, *E. faecalis* is more prevalent than *E. hirae* [13,19]; however, instances in which *E. hirae* was more dominant than *E. faecalis* have been reported [14,28]. In an urban watershed, *E. mundtii* was recently found to be the most dominant species at 32% [29] and in hospital wastewater, *E. faecalis* was the second most dominant species after *E. faecium* [30]. *E. faecalis* is predominantly found in hospital-derived samples, whereas *E. hirae* is more common in livestock manure samples [11,24]. *E. faecium* and *E. faecalis* are primarily associated with the human environment, whereas *E. hirae* is frequently isolated from cattle feces and related wastewater [24]. The higher ratio of *E. hirae* than of *E. faecalis* in this study is likely because four of the 11 WWTPs were associated with livestock manure treatment facilities. 

Additionally, we identified variations in the resistance patterns among different *Enterococcus* spp., with some species exhibiting higher levels of resistance to certain classes of antibiotics. In particular, *E. faecalis* had an antibiotic resistance rate of 100%, which was significantly higher than those of *E. faecium* and *E. hirae*. This is likely attributable to the high quinupristin/dalfopristin resistance rate of 95.5% observed in *E. faecalis*, which is believed to result from intrinsic resistance, as demonstrated in previous studies [25]. Its MDR rate was also more than twice that of the other two species. However, recent studies have reported that notwithstanding variations among antibiotics, *E. faecium* generally exhibits higher antibiotic resistance rates than *E. faecalis* [11,29,31]. In particular, for *Enterococcus* strains isolated from hospitals, the MDR rate of *E. faecium* was 75.6%, which was significantly higher than the 21.9% observed for *E. faecalis* [32]. In addition, *E. faecium* showed higher rates of resistance to ampicillin and aminoglycoside antibiotics such as gentamicin and streptomycin than *E. faecalis*. However, in the present study, among the antibiotics with statistically significant differences in resistance rates among *Enterococcus* spp., *E. faecalis* exhibited higher rates of resistance to chloramphenicol, erythromycin, tylosin tartrate, streptomycin, and quinupristin/dalfopristin, whereas *E. faecium* showed a higher rate of resistance to ciprofloxacin. Although the ampicillin antibiotic resistance rate of hospital-derived *E. faecium* is known to exceed 90%, this study, focusing on *E. faecium* isolated from WWTPs, found the rate to be less than 5%. This result aligns closely with the findings of the National Antibiotic Usage and Resistance Monitoring Reports (2022–2023) [33,34], which reported an ampicillin resistance rate of *E. faecium* isolated from livestock feces at approximately 5.5–8.8%. This indicates that antibiotic resistance patterns vary according to the *Enterococcus* spp. and strain of origin, highlighting the need for species-level identification and targeted approaches to better understand the dynamics and public health implications. 

Recently, WWTPs have been identified as key areas for the spread of vancomycin-resistant enterococci [19], leading to increased research in this area. However, *Enterococcus* strains isolated from clinical samples exhibit high susceptibility to linezolid and vancomycin [11,35], a pattern that has also been observed in strains isolated from WWTPs. Although some studies have reported vancomycin resistance rates as high as 62% in *Enterococcus* strains isolated from WWTPs [26], in most cases, including our study, the vancomycin resistance rate in WWTPs was less than 1% [22,28]. Recent studies have indicated that vancomycin-resistant *Enterococcus* spp. rates range from less than 1% in some settings to as high as 55% in others [36]. In addition, a meta-analysis found an average vancomycin resistance rate of approximately 4.3% across multiple studies, indicating significant heterogeneity depending on geographical location and clinical setting [37]. This variation is influenced by factors such as antibiotic usage patterns, infection control practices, and local microbial ecology. 

As the incidence of antibiotic resistance in *E. faecalis* increases worldwide, underscoring its role as a potential opportunistic pathogen, there is an urgent need to develop alternative treatments and new antibiotic agents to effectively combat resistant strains [38]. Recent meta-analysis results indicated that daptomycin and tigecycline have potential as treatment options for *E. faecalis* owing to their low resistance rates in Europe and Australia [37]. Based on the results of this study, the usual antibiotics for treatment of *E. faecalis* infection such as ampicillin remain susceptible. 

The most frequently detected ARGs were *tetM* and *tetL*, which are associated with tetracycline resistance, and *ermB*, which is associated with macrolide resistance. Regardless of the strain origin, *tetM* and *ermB* are commonly observed in *Enterococcus* spp. isolated from various sources [10,24]. Notably, these genes were most frequently detected in *E. faecalis* compared with other species, which is consistent with previous research results [15]. The plasmid-mediated quinolone resistance gene, *qnrS*, was significantly more prevalent in the effluent than in the influent. *qnrS* facilitates the spread of resistance among bacteria in various environments, including WWTPs [39]. In addition, some studies have indicated that this ARG is often not fully eliminated from WWTPs, leading to higher concentrations in effluents [40]. Among the genes associated with oxazolidinone resistance, *optrA* was detected at high frequencies in *E. faecalis*, whereas *poxtA* was predominantly detected in *E. faecium*. In a previous study conducted in China, *E. faecalis* isolated from poultry and pig fecal samples showed a higher detection rate for *optA* than other species [10]. 

Sub-inhibitory concentrations of antibiotics in wastewater can promote the growth of resistant bacteria, facilitate gene transfer, and enhance biofilm formation in wastewater treatment tanks or pipelines [18,22]. Elevated levels of antibiotics and other pharmaceuticals in the environment create favorable conditions for the selection of antibiotic resistance and serve as significant hotspots for the horizontal gene transfer of ARGs, promoting the evolution of resistance [2]. In this study, ciprofloxacin was detected at higher concentrations than the other residual antibiotics in both the influent and effluent of the 11 WWTPs. Additionally, the ciprofloxacin resistance rate was significantly higher in the effluent than in the influent. Although the residual ciprofloxacin concentration was not high, continuous exposure to WWTPs may have contributed to the increase in antibiotic resistance rates in enterococci [41]. In the case of ampicillin and gentamicin, despite an increase in antibiotic resistance rates after passing through the WWTP, no residual antibiotics were detected. This suggests that the presence of ARB in wastewater does not necessarily indicate the presence of the corresponding antibiotics [42]. Possible reasons for the presence of resistant bacteria, even in the absence of detectable residual antibiotics, include prior exposure to antibiotics before entering the WWTP, horizontal gene transfer of ARGs, and the development of resistance mechanisms due to environmental stress factors such as high concentrations of organic matter within the WWTP.

Biofilm formation, a significant virulence factor in enterococci, varies globally and is influenced by the species, host, and environmental conditions. It not only increases antibiotic resistance but also poses a risk factor for the spread of hospital-acquired infections. Biofilm-forming bacteria are responsible for more than 65% of nosocomial infections and 80% of bacterial infections, with *Enterococcus* biofilm production being a key pathogenic trait, particularly in urology [43,44]. However, the biofilm formation rate of *Enterococcus* spp. isolated from WWTPs was relatively low at approximately 20%, with most strains forming only weak biofilms. Although recent research has shown that vancomycin-resistant strains exhibit greater biofilm formation than non-resistant strains [9], none of the vancomycin-resistant strains formed biofilms. In previous studies, bacteria in biofilms formed in effluents exhibited higher resistance to all tested antibiotics than those found in influent biofilms [45]. In contrast, in the present study, we found that strong biofilm formation did not consistently correlate with higher resistance rates to multiple antibiotics.

The final effluent discharged into the environment contains MDR *Enterococcus* spp., posing a potential health risk to receiving aquatic systems, as these bacteria may be transmitted to humans and animals exposed to the contaminated water [2]. The antibiotic resistance profiles of *Enterococcus* spp. were found to be very similar when comparing samples from a WWTP with those collected at a site located 500 m downstream of the WWTP [13]. The results of this study showed no difference in antibiotic resistance rates between the influent and effluent, indicating that ARB were not completely eliminated during wastewater treatment. As we examined the influent and effluent of the combination of 11 WWTPs, it was only possible to determine the overall trend of how antibiotic resistance rates changed as they passed through the WWTPs. However, because each WWTP employs different water treatment processes, the efficiency of antibiotic resistance removal likely varies. Therefore, future research is necessary to explore how antibiotic resistance changes based on the specific treatment methods employed by individual WWTPs. Furthermore, because surveillance studies are crucial for identifying shifts in the resistance patterns of key pathogens [11], continued research on major pathogens other than *Enterococcus* spp. in WWTPs, which serve as the primary route to the natural environment, is essential. 

## 4. Materials and Methods

### 4.1. Isolation and Identification of Enterococcus *spp.*

In 2022, influent and effluent samples were collected from 11 WWTPs in South Korea. After thorough homogenization, 100 µL of each sample was inoculated onto kanamycin esculin azide agar (MB Cell, Seoul, Republic of Korea) to obtain pure strains of *Enterococcus* spp. The plates were then incubated at 36 °C for 24 h, and grayish-black colonies with black halos were selected. These colonies were transferred to *Enterococcus*-selective agar (Sigma-Aldrich, Saint Louis, MO, USA) for further isolation and incubated at 36 °C for 24 h. Pink and dark red colonies were identified. For species confirmation, the selected colonies were transferred to tryptic soy agar (BD Difco^TM^, Franklin Lakes, NJ, USA), incubated, and analyzed using a matrix-assisted laser desorption/ionization time-of-flight mass spectrometry (MALDI-TOF MS) system (Bruker Daltonics, Billerica, MA, USA), which provided the final identification of *Enterococcus* spp. [46]. 

### 4.2. Antibiotic Susceptibility Testing

The antibiotic susceptibility of the *Enterococcus* isolates was assessed using the microdilution method, a widely adopted approach for evaluating phenotypic resistance. Bacterial suspensions were standardized to a 0.5 McFarland turbidity and inoculated into a commercially available 96-well microtiter plate (KRVP2F, Daejeon, Republic of Korea), which contained 16 antibiotic agents and is commonly utilized in the livestock industry. After a 24 h incubation at 35 °C, the minimum inhibitory concentration (MIC) was determined either by visual inspection or using an automated reader. The automated reader streamlined the interpretation of the results by accurately detecting microbial growth and ensuring consistent data recording. MIC breakpoints for the 16 antibiotic agents were interpreted according to the established guidelines of the Clinical Laboratory Standard Institute [47], the European Committee on Antimicrobial Susceptibility Testing [48], and the Danish Integrated Antimicrobial Resistance Monitoring and Research Programme [49]. The proportion of antibiotic-resistant strains was calculated as the number of strains with MIC values exceeding the respective breakpoint for each antibiotic divided by the total number of isolates tested. The concentration range of each antibiotic tested and the breakpoints for the resistance readings are shown in Supplementary Table S1.

### 4.3. Identification of Antibiotic Resistance Genes

Genomic DNA was extracted using a DNeasy Plant Mini Kit (Qiagen, Valencia, CA, USA), following the manufacturer’s protocol. To detect the presence of ARGs, polymerase chain reaction (PCR) testing was conducted targeting specific genes. The genes investigated included tetracycline resistance genes (*tetA*, *tetB*, *tetM*, and *tetL*), macrolide resistance genes (*ermA*, *ermB*, and *ermC*), fluoroquinolone resistance genes (*qnrA*, *qnrB*, *qnrS*, and *qepA*), the phenicol resistance gene (*catA*), oxazolidinone resistance genes (*optrA* and *poxtA*), and glycopeptide resistance genes (*vanA*, *vanB*, and *vanHM*) [50,51,52]. The primer sequences and PCR conditions are presented in Supplementary Table S2. Additionally, plasmids were generated and used as positive controls, utilizing bacterial strains confirmed to be positive for each respective ARG. 

### 4.4. Analysis of Biofilms

Biofilm formation assays were carried out following established protocols [51,52,53], with minor adjustments. Bacterial cultures were grown on Mueller–Hinton agar and standardized to a density equivalent of 0.5 McFarland units in distilled water. A 10-µL aliquot of each bacterial suspension was diluted 1:20 in 190 µL Luria–Bertani broth in 96-well plates. The plates were then incubated at 37 °C for 24 or 48 h. After incubation, non-adherent bacteria were removed by washing the wells three times with distilled water. The adherent cells were fixed by adding 200 µL methanol per well. After air-drying the plates, the remaining biofilms were stained with 0.1% crystal violet solution for 20 min. The wells were rinsed thoroughly with distilled water and left to dry. To quantify biofilm formation, 200 µL ethanol was added to each well to resuspend the crystal violet bound to the cells, and the optical density (OD) was measured at 550 nm. The biofilm formation levels were categorized as negative, weak, moderate, or strong based on the OD readings. The cutoff value (ODc) was calculated as the mean OD of the negative control plus three standard deviations. All experiments were conducted in triplicate, and the average results were recorded. Following a previously described method [51,53,54] with minor modifications, OD values from the negative control, measured at 595 nm, were used to determine the ODc. Biofilm production was classified as follows: OD < ODc for non-biofilm producers; ODc < OD < 2× ODc for weak producers; 2× ODc < OD < 4× ODc for moderate producers; and OD > 4× ODc for strong producers.

### 4.5. Detection of Antibiotic Residues

A total of 26 antibiotics were examined for residual presence. The antibiotics tested were amoxicillin, ampicillin, cefepime, cefoxitin, ceftazidime, ceftiofur, chloramphenicol, ciprofloxacin, clavulanic acid, erythromycin, florfenicol, linezolid, meropenem, nalidixic acid, salinomycin, sulfamethoxazole, sulfisoxazole, tetracycline, tigecycline, trimethoprim, tylosin, vancomycin, kanamycin, streptomycin, gentamicin, and colistin. To analyze antibiotic residues in the wastewater samples, 500 mL of each sample was first filtered through a 0.2-μm polyvinylidene difluoride membrane filter. Next, 900 µL of the filtered solution was transferred into amber autosampler vials, followed by the addition of 100 µL of 1% acetic acid solution, 40 mg/mL ethylenediaminetetraacetic acid disodium salt dihydrate, and 10 µL of 10 ng/mL isotopically labeled internal standards. The pretreated sample (200 μL) was subsequently analyzed using high-performance liquid chromatography combined with tandem mass spectrometry [55,56]. The findings on the presence and average concentrations of antibiotic residues from across the 11 WWTPs are reported in the Results Section.

### 4.6. Statistical Analysis

A chi-square test was conducted using R software (version 4.3.0) to assess the statistical significance of differences in antibiotic resistance rates between the influent and effluent and evaluate the association between the antibiotic resistance phenotype and corresponding ARGs. All statistical analyses were performed at a significance level of *p* < 0.05.

## 5. Conclusions

In conclusion, this study demonstrated that the antibiotic resistance rate of *Enterococcus* spp. did not significantly change after treatment in WWTPs and the failure to fully eliminate these resistant bacteria allows them to be discharged into aquatic environments, posing ongoing public health risks. These findings underscore the need for continuous monitoring and improvement of WWTP processes to enhance the removal of antibiotic-resistant bacteria. The detection of high resistance in species such as *E. faecium, E. hirae,* and *E. faecalis* further emphasizes the need to identify and mitigate hotspots of multidrug-resistant bacteria. Given the variability in the treatment efficiency across different WWTPs, future studies should focus on the effects of specific treatment methods on antibiotic resistance. Furthermore, broader surveillance of major pathogens beyond *Enterococcus* spp. is critical, because WWTPs are key conduits for these bacteria in the natural environment. A comprehensive approach that includes prudent antibiotic use, development of new antimicrobial agents, robust infection control, and global surveillance is essential to combat the spread of antibiotic resistance and safeguard public health.

## Figures and Tables

**Table 1:** Comparison of antibiotic resistance rates in wastewater before and after passing through WWTPs and antibiotic resistance rates among different *Enterococcus* spp.

	WWTPs		Three Major *Enterococcus* spp.		
	Influents	Effluents	*E. faecium*	*E. faecalis*	*E. hirae*
Antibiotic resistance rate (resistant to at least one antibiotic)	59.9% (287/479)	62.8% (204/325)	62% (308/496)	100% (44/44)	52% (134/256)
Multidrug resistance rate (resistant to three or more classes of antibiotics)	20.9% (100/479)	18.2% (59/325)	20% (99/496)	43.2% (19/44)	15.2% (39/256)

**Table 2:** Comparison of biofilm formation abilities of *Enterococcus* isolates in the influents and effluents of the WWTPs.

	None	Weak	Moderate	Strong
Influents	76.4% (366/479)	18.2% (87/479)	5.0% (24/479)	0.4% (2/479)
Effluents	79.4% (258/325)	10.8% (35/325)	8.0% (26/325)	1.8% (6/325)

## Data Availability

The original contributions presented in the study are included in the article/supplementary material, and further inquiries can be directed to the corresponding author.

## Supplementary Materials

The following supporting information can be downloaded at: https://www.mdpi.com/article/10.3390/antibiotics13111072/s1, Table S1: MIC breakpoints for antibiotic susceptibility testing, Table S2: List of primers and PCR conditions used in this study.
