# Supplementary material for: Comprehensive Study of Antibiotic Resistance in Enterococcus spp.: Comparison of Influents and Effluents of Wastewater Treatment Plants
Source: Antibiotics (Basel). 2024 Nov 11;13(11):1072. doi: 10.3390/antibiotics13111072 (PMC11590936; doi:10.3390/antibiotics13111072)
Supplement: Supplementary file 1 [file antibiotics-13-01072-s001.zip › Supplementary Material_Table S1.pdf]

Table S1 – MIC breakpoints for antibiotic susceptibility testing

Antibiotic subclass	Antibiotic agent	Range tested (µg/mL)	Breakpoint (µg/mL)
Aminopenicillin	ampicillin	1-16	≥16
Phenicols	chloramphenicol	2-32	≥32
	florfenicol	2-32	≥16
Fluoroquinolone	ciprofloxacin	0.25-16	≥4
Lipopeptides	daptomycin	0.5-32	≥8
Macrolides	erythromycin	1-64	≥8
	tylosin tartrate	1-64	≥32
Aminoglycosides	gentamycin	128-2048	≥1024
	kanamycin	128-2048	≥1024
	streptomycin	128-2048	≥1024
Oxazolidinones	linezolid	0.5-16	≥8
Streptogramins	quinupristin/dalfopristin	1-32	≥4
Tetracylclines	tetracycline	2-128	≥16
Glycylcyclines	tigecyline	0.12-2	≥0.5
Glycopeptide	vancomycin	2-32	≥32
Others	salinomycin	2-32	≥8
