# Supplementary material for: Comprehensive Study of Antibiotic Resistance in Enterococcus spp.: Comparison of Influents and Effluents of Wastewater Treatment Plants
Source: Antibiotics (Basel). 2024 Nov 11;13(11):1072. doi: 10.3390/antibiotics13111072 (PMC11590936; doi:10.3390/antibiotics13111072)
Supplement: Supplementary file 1 [file antibiotics-13-01072-s001.zip › Supplementary Material_Table S2.pdf]

Table S2 – List of primers and PCR conditions used in this study.

| Class | Target | Primer Name | Primer Sequence | Size (pb) | *Annealing* (T) | Ref. |
|---|---|---|---|---|---|---|
| Tetracycline | *tetA* | tetA-F | 5′-GGCACCGAATGCGTATGAT-3′ | 480 | 54°C | [49] |
| | | tetA-R | 5′-AAGCGAGCGGGTTGAGAG-3′ | | | |
| | *tetB* | tetB-F | 5′-CTCAGTATTCCAAGCCTTTC-3′ | 416 | 54°C | [49] |
| | | tetB-R | 5′-CTAAGCACTTGTCTCCTGTT-3′ | | | |
| | *tetM* | tetM-F | 5′-GTTAAATAGTGTTCTTGGAG-3′ | 657 | 53°C | [49] |
| | | tetM-R | 5′-CTAAGATATGGCTCTAACAA-3′ | | | |
| | *tetL* | tetL- F | 5′-ATAAATTGTTTCGGGTCGGTAAT-3′ | 1077 | 50°C | [49] |
| | | tetL-R | 5′-AACCAGCCAACTAATGACAATGAT-3′ | | | |
| Macrolides | *ermA* | ermA-F | 5′-TCTAAAAAGCATGTAAAAGAA-3′ | 645 | 52°C | [49] |
| | | ermA-R | 5′-CTTCGATAGTTTATTAATATTAGT-3′ | | | |
| | *ermB* | ermB-F | 5′-GAAAAGGTACTCAACCAAATA-3′ | 639 | 52°C | [49] |
| | | ermB-R | 5′-AGTAACGGTACTTAAATTGTTTAC-3′ | | | |
| | *ermC* | ermC-F | 5′-GCTAATATTGTTTAAATCGTCAATTCC-3′ | 572 | 58°C | [48] |
| | | ermC-R | 5′-GGATCAGGAAAAGGACATTTTAC-3′ | | | |
| Fluoroquinolone | *qnrA* | qnrA-F | 5′-GCCCGCTTCTACAATCAAGT-3′ | 347 | 60°C | [49] |
| | | qnrA-R | 5′-GGCAGCACTATTACTCCCAAG-3′ | | | |
| | *qnrB* | qnrB-F | 5′-TATGGCTCTGGCACTCGTT-3′ | 193 | 60°C | [49] |
| | | qnrB-R | 5′-GCATCTTTCAGCATCGCAC-3′ | | | |
| | *qnrS* | qnrS-F | 5′-TCGGCACCACAACTTTTCAC-3′ | 255 | 60°C | [49] |
| | | qnrS-R | 5′-TCACACGCACGGAACTCTAT-3′ | | | |
| | *qepA* | qepA-F | 5′-TCTACGGGCTCAAGCAGTTG-3′ | 312 | 55°C | [49] |
| | | qepA-R | 5′-ACAGCGAACCGATGACGAAG-3′ | | | |
| Phenicols | *catA* | cat-F | 5′-GGATATGAAATTTATCCCTC-3′ | 486 | 54°C | [49] |
| | | cat-R | 5′-CAATCATCTACCCTATGAAT-3′ | | | |
| Oxazolidinones | *optrA* | optrA-F | 5′-AGGTGGTCAGCGAACTAA-3′ | 1395 | 48°C | [49] |
| | | optrA-R | 5′-ATCAACTGTTCCCATTCA-3′ | | | |
| | *poxtA* | poxtA-F | 5′-GGTGGATTTACCGACACCGT-3′ | 943 | 50°C | [49] |
| | | poxtA-R | 5′-GACCAGTGGAAATGCCCGTA-3′ | | | |
| Glycopeptide | *vanA* | vanA-F | 5′-GCGCGGTCCACTTGTAGATA-3′ | 314 | 54°C | [49] |
| | | vanA-R | 5′-TGAGCAACCCCCAAACAGTA-3′ | | | |
| | *vanB* | vanB-F | 5′-AGACATTCCGGTCGAGGAAC-3′ | 220 | 54°C | [49] |
| | | vanB-R | 5′-GCTGTCAATTAGTGCGGGAA-3′ | | | |
| | *vanHM* | vanHM-F | 5′-CAGCGTGGGGCACAAGTCTGA-3′ | 377 | 55°C | [50] |
| | | vanHM-R | 5′-TGCCGTACGCCAACACGTGA-3′ | | | |
